# Supplementary material for: Analog quantum simulation of coupled electron-nuclear dynamics in molecules
Source: Chem Sci. 2025 Sep 16;16(41):19423–35. doi: 10.1039/d5sc04076k (PMC12459289; doi:10.1039/d5sc04076k)
Supplement: SC-016-D5SC04076K-s001 [file SC-016-D5SC04076K-s001.pdf]

Supplementary Information (SI) for Chemical Science.
This journal is © The Royal Society of Chemistry 2025

Electronic Supplementary Information for "Analog Quantum Simulation of Coupled Electron-Nuclear Dynamics in Molecules"

Jong-Kwon Ha[1] and Ryan J. MacDonell[1, 2, *]

[1] *Department of Chemistry, Dalhousie University, 6243 Alumni Cres, Halifax, NS B3H 4R2, Canada*

[2] *Department of Physics and Atmospheric Science, Dalhousie University, 1453 Lord Dalhousie Dr, Halifax, NS B3H 4R2, Canada*

(Dated: August 12, 2025)

I. ELECTRONIC INTEGRALS

The electronic integral terms in Eq. (3) (one- and two-electron integrals, and the first and second order orbital vibronic couplings) depend on the vibrational coordinates, and are given by

$$h_{pq}(\mathbf{Q}) = \delta_{\sigma_p\sigma_q} \int \mathrm{d}\mathbf{r}\, \phi_p^*(\mathbf{r};\mathbf{Q}) \Big(-\frac{\nabla^2}{2} + v_{\mathrm{en}}(\mathbf{r},\mathbf{Q}) \Big) \phi_q(\mathbf{r};\mathbf{Q}), \tag{S1}$$

$$v_{pqrs}(\mathbf{Q}) = \delta_{\sigma_p\sigma_r}\delta_{\sigma_q\sigma_s} \int \mathrm{d}\mathbf{r} \int \mathrm{d}\mathbf{r}'\, \phi_p^*(\mathbf{r};\mathbf{Q})\phi_q^*(\mathbf{r}';\mathbf{Q}) \frac{1}{|\mathbf{r}-\mathbf{r}'|} \phi_r(\mathbf{r};\mathbf{Q})\phi_s(\mathbf{r}';\mathbf{Q}), \tag{S2}$$

$$d_{\nu,pq}(\mathbf{Q}) = \delta_{\sigma_p\sigma_q} \int \mathrm{d}\mathbf{r}\, \phi_p^*(\mathbf{r};\mathbf{Q}) \frac{\partial}{\partial Q_\nu} \phi_q(\mathbf{r};\mathbf{Q}), \tag{S3}$$

$$g_{\nu,pq}(\mathbf{Q}) = \delta_{\sigma_p\sigma_q} \int \mathrm{d}\mathbf{r}\, \phi_p^*(\mathbf{r};\mathbf{Q}) \frac{\partial^2}{\partial Q_\nu^2} \phi_q(\mathbf{r};\mathbf{Q}). \tag{S4}$$

The electron-nuclear potential $v_{\mathrm{en}}(\mathbf{r},\mathbf{Q})$ and the nuclear-nuclear potential $V_{\mathrm{nn}}(\mathbf{Q})$ are given in terms of Cartesian nuclear positions $\mathbf{R}_\alpha$ for nucleus α,

$$v_{\mathrm{en}}(\mathbf{r},\mathbf{Q}) = -\sum_\alpha \frac{Z_\alpha}{|\mathbf{r}-\mathbf{R}_\alpha|}, \tag{S5}$$

$$V_{\mathrm{nn}}(\mathbf{Q}) = \sum_{\alpha\beta} \frac{Z_\alpha Z_\beta}{|\mathbf{R}_\alpha-\mathbf{R}_\beta|}, \tag{S6}$$

where Z_α is the charge of nucleus α. The nuclear internal coordinates and the vector of all Cartesian coordinates are related by a unitary transformation $\mathbf{Q} = \mathbf{U}\mathbf{M}^{1/2}\mathbf{R}$, where

$$\mathbf{R} = \begin{pmatrix} \mathbf{R}_1 \\ \mathbf{R}_2 \\ \vdots \\ \mathbf{R}_n \end{pmatrix} \tag{S7}$$

for a molecule with n atoms.

II. TWO-ELECTRON SHIN-METIU MODEL HAMILTONIAN

The one-dimensional, two-electron Shin-Metiu model Hamiltonian has two fixed ions, a moving ion between them, and two electrons in one-dimensional space [1, 2]:

$$\hat{H}(r_1,r_2,R) = -\frac{1}{2M}\frac{\partial^2}{\partial R^2} + V_{\mathrm{nn}}(R) + \hat{h}_{1\mathrm{e}}(r_1,R) + \hat{h}_{1\mathrm{e}}(r_2,R) + v_{\mathrm{ee}}(r_1,r_2). \tag{S8}$$

---

* rymac@dal.ca

TABLE S.I. Taylor expansion coefficients of the nuclear position R for the electron integrals

$v(R) \approx v_0 + v_1 R$	h_{aa}	h_{bb}	h_{ab}	v_{aaaa}	v_{bbbb}	v_{abab}	v_{aaab}	v_{abbb}	v_{aabb}
v_0	−2.66	−2.66	−0.0046	0.2236	0.2236	0.1652	0.0001	0.0001	0.0000015
v_1	0.2	−0.2	0.0	0.00044	−0.00044	0.0	0.0	0.0	0.0

Here, r_i represents the position of the electron i, R is the position of the moving ion, and $M = 1836.0$ a.u. is the mass of the moving ion. In the above equation, we replaced the original Coulomb repulsion exerted on the moving ion by the fixed ions by a harmonic potential,

$$V_{\mathrm{nn}}(R) = \frac{1}{2}kR^2, \tag{S9}$$

with a spring constant $k = 4.0$ a.u. The one-electron Hamiltonian $\hat{h}_{1\mathrm{e}}$ [1] is defined as

$$\hat{h}_{1\mathrm{e}}(r, R) = -\frac{1}{2}\frac{\partial^2}{\partial r^2} - \frac{\mathrm{erf}\left(\frac{|R-r|}{C_{\mathrm{c}}}\right)}{|R-r|} - \frac{\mathrm{erf}\left(\frac{|r-\frac{L}{2}|}{C_{\mathrm{r}}}\right)}{|r-\frac{L}{2}|} - \frac{\mathrm{erf}\left(\frac{|r+\frac{L}{2}|}{C_{\mathrm{l}}}\right)}{|r+\frac{L}{2}|}, \tag{S10}$$

and the electron-electron interaction [2] is given by

$$v_{\mathrm{ee}}(r_1, r_2) = \frac{\mathrm{erf}\left(\frac{|r_1-r_2|}{C_{\mathrm{e}}}\right)}{|r_1-r_2|}, \tag{S11}$$

where $L = 5.4$ a.u. is the distance between fixed ions. $C_{\mathrm{l}} = C_{\mathrm{r}} = 0.3$ a.u., and $C_{\mathrm{c}} = 0.6$ a.u. are soft Coulomb parameters for the fixed ions and the moving ion, respectively, and $C_{\mathrm{e}} = 5.0$ a.u. is a soft Coulomb parameter for electron-electron repulsion. The origin is set to the mid-point of the two fixed ions. All parameters in the model Hamiltonian are chosen such that the model dynamics show an oscillation in the occupation numbers of spin-orbitals within the time range of 2500 a.u. to exhibit a vibronic effect, while keeping the Taylor expansion order for electron integrals and nuclear basis set small for a feasible classical simulation.

We selected the two diabatic orbitals η_a and η_b with spin-up and spin-down configurations as a spin-orbital basis set, obtained from two adiabatic orbitals ψ_l,. The adiabatic orbitals are the eigenstates of the Schrödinger equation $\hat{h}_{1\mathrm{e}}\psi_l = e_l\psi_l$ for the one-electron Shin-Metiu model [1]. The electron integrals of the diabatic orbitals and the nuclear bound potential are shown in Fig. S1.

The diabatic orbitals [Figs. S1(a) and S1(b)] are very localized and not significantly changed by the nuclear motion due to the strong nonadiabatic character of the model system with the chosen parameters [1]. The magnitude of changes in some integrals is also small. Therefore, we can approximate the electron integrals of diabatic orbitals up to the first order Taylor expansion of the nuclear position ($v(R) \approx v_0 + v_1 R$) where $R = Q/\sqrt{M}$, and $\hat{Q} = (\hat{b}^\dagger + \hat{b})/\sqrt{2}$ with ladder operators $\hat{b}^\dagger$ and $\hat{b}$ of the single mode. The expansion coefficients for the electron integrals are given in Table S.I.

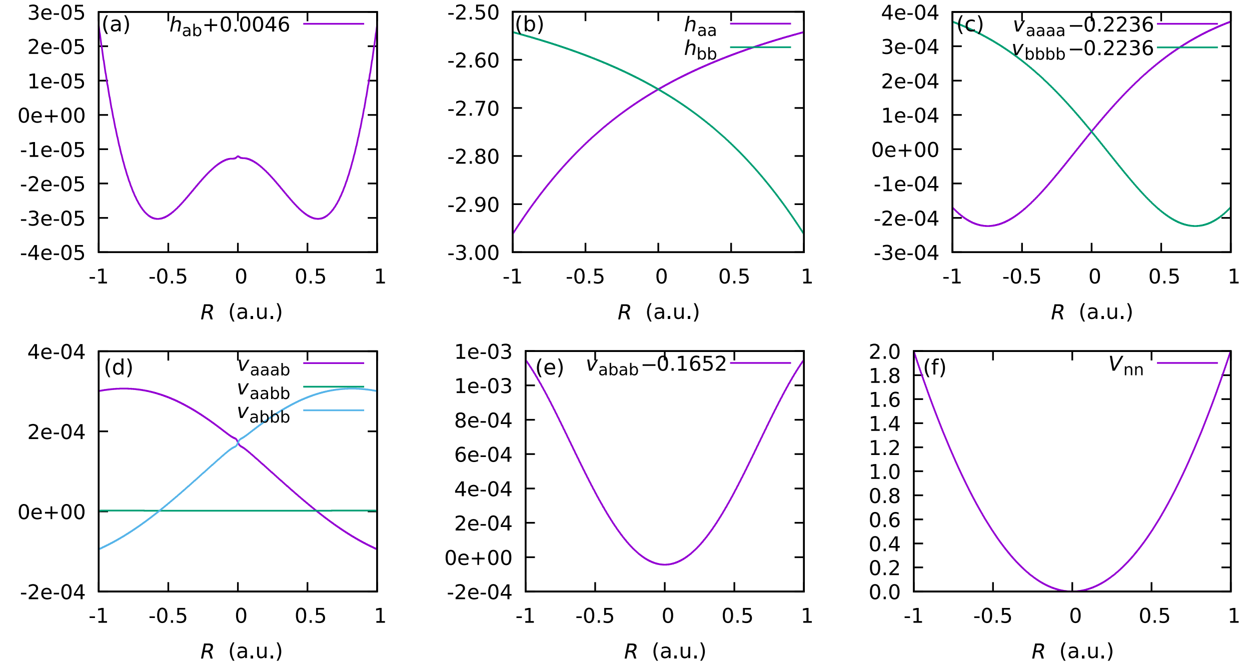

FIG. S1. The one-/two-electron integrals and the nuclear bound potential. (a)–(b) one-electron integrals h_{pq}. (c)–(e) two-electron integrals v_{pqrs}. (f) nuclear bound potential $V_{\rm nn}$.

III. HAMILTONIAN MAPPING

As a result of the Jordan-Wigner mapping with the four spin orbitals, the two-electron Shin-Metiu model Hamiltonian is mapped to

$$
\begin{aligned}
\hat{H}_{\rm mol} = & -\frac{1}{2M}\frac{\partial^2}{\partial R^2} + \frac{1}{2}kR^2 + h_{aa} + h_{bb} + \frac{1}{4}(v_{aaaa} + v_{bbbb} + 4v_{abab} - 2v_{aabb}) \\
& - \left(\frac{h_{aa}}{2} + \frac{1}{4}(v_{aaaa} + 2v_{abab} - v_{aabb})\right)(\hat{Z}_1 + \hat{Z}_3) - \left(\frac{h_{bb}}{2} + \frac{1}{4}(v_{bbbb} + 2v_{abab} - v_{aabb})\right)(\hat{Z}_2 + \hat{Z}_4) \\
& + \frac{1}{4}v_{aaaa}\hat{Z}_1\hat{Z}_3 + \frac{1}{4}v_{bbbb}\hat{Z}_2\hat{Z}_4 + \frac{1}{4}v_{abab}(\hat{Z}_1\hat{Z}_4 + \hat{Z}_2\hat{Z}_3) + \frac{1}{4}(v_{abab} - v_{aabb})(\hat{Z}_1\hat{Z}_2 + \hat{Z}_3\hat{Z}_4) \\
& + \left(\frac{h_{ab}}{2} + \frac{1}{4}(v_{aaab} + v_{abbb})\right)(\hat{X}_1\hat{X}_2 + \hat{Y}_1\hat{Y}_2 + \hat{X}_3\hat{X}_4 + \hat{Y}_3\hat{Y}_4) \\
& + \frac{1}{4}v_{aabb}(\hat{X}_1\hat{X}_2\hat{X}_3\hat{X}_4 + \hat{Y}_1\hat{Y}_2\hat{Y}_3\hat{Y}_4 + \hat{X}_1\hat{X}_2\hat{Y}_3\hat{Y}_4 + \hat{Y}_1\hat{Y}_2\hat{X}_3\hat{X}_4) \\
& - \frac{1}{4}v_{aaab}(\hat{Z}_1\hat{X}_3\hat{X}_4 + \hat{Z}_1\hat{Y}_3\hat{Y}_4 + \hat{X}_1\hat{X}_2\hat{Z}_3 + \hat{Y}_1\hat{Y}_2\hat{Z}_3) \\
& - \frac{1}{4}v_{abbb}(\hat{Z}_2\hat{X}_3\hat{X}_4 + \hat{Z}_2\hat{Y}_3\hat{Y}_4 + \hat{X}_1\hat{X}_2\hat{Z}_4 + \hat{Y}_1\hat{Y}_2\hat{Z}_4).
\end{aligned} \tag{S12}
$$

After truncating to first-order terms with the parameters in the Table S.I, the final coupled multi-qubit-boson (cMQB) Hamiltonian used for the dynamics can be written as

$$\begin{aligned}\hat{H}_{\mathrm{cMQB}} = \omega\hat{b}^\dagger\hat{b} + V_0^0 + V_1^0(\hat{b}+\hat{b}^\dagger) + \left(V_0^1 + V_1^1(\hat{b}+\hat{b}^\dagger)\right)(\hat{Z}_1+\hat{Z}_3) + \left(V_0^2 + V_1^2(\hat{b}+\hat{b}^\dagger)\right)(\hat{Z}_2+\hat{Z}_4)\\ + \left(V_0^3 + V_1^3(\hat{b}+\hat{b}^\dagger)\right)\hat{Z}_1\hat{Z}_3 + \left(V_0^4 + V_1^4(\hat{b}+\hat{b}^\dagger)\right)\hat{Z}_2\hat{Z}_4\\ + V_0^5(\hat{Z}_1\hat{Z}_4+\hat{Z}_2\hat{Z}_3) + V_0^6(\hat{Z}_1\hat{Z}_2+\hat{Z}_3\hat{Z}_4)\\ + V_0^7(\hat{X}_1\hat{X}_2+\hat{Y}_1\hat{Y}_2+\hat{X}_3\hat{X}_4+\hat{Y}_3\hat{Y}_4)\\ + V_0^8(\hat{X}_1\hat{X}_2\hat{X}_3\hat{X}_4+\hat{Y}_1\hat{Y}_2\hat{Y}_3\hat{Y}_4+\hat{X}_1\hat{X}_2\hat{Y}_3\hat{Y}_4+\hat{Y}_1\hat{Y}_2\hat{X}_3\hat{X}_4)\\ + V_0^9(\hat{Z}_1\hat{X}_3\hat{X}_4+\hat{Z}_1\hat{Y}_3\hat{Y}_4+\hat{X}_1\hat{X}_2\hat{Z}_3+\hat{Y}_1\hat{Y}_2\hat{Z}_3)\\ + V_0^{10}(\hat{Z}_2\hat{X}_3\hat{X}_4+\hat{Z}_2\hat{Y}_3\hat{Y}_4+\hat{X}_1\hat{X}_2\hat{Z}_4+\hat{Y}_1\hat{Y}_2\hat{Z}_4),\end{aligned} \tag{S13}$$

where $\omega = \sqrt{k/M}$ and V_i^I corresponds to the i-th order net expansion coefficients of Q for the I-th group of Pauli strings.

IV. COUPLED MULTI-QUBIT-BOSON SIMULATION

For the dynamics simulation and the calculation of the density shown in Fig. 3, we used 20 harmonic oscillator eigenfunctions as a bosonic basis set.

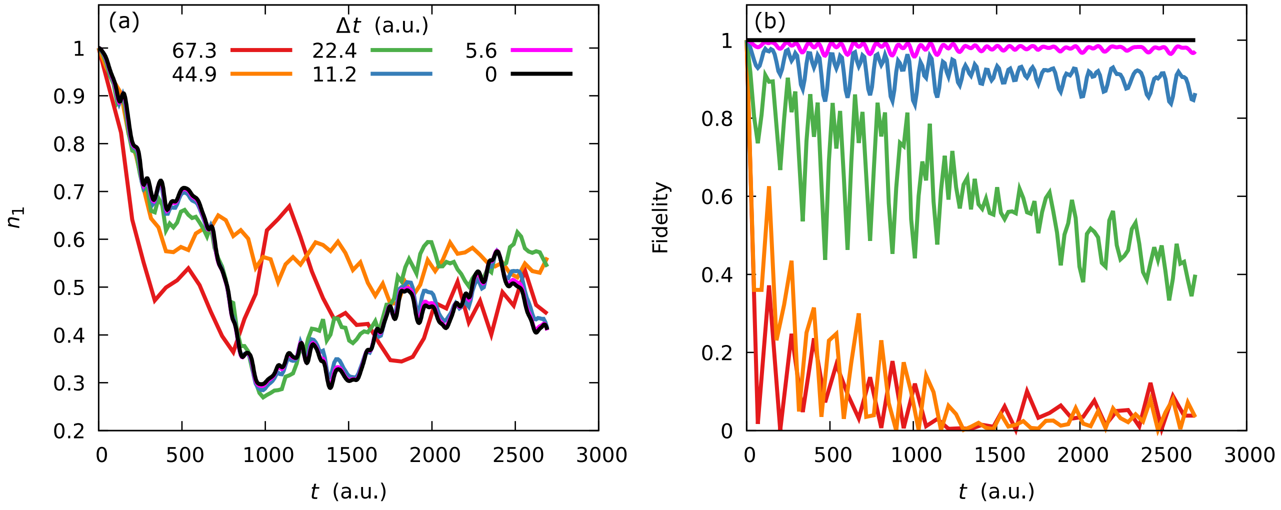

FIG. S2. Time-evolution of orbital occupation number and fidelity. The black line ($\Delta t = 0$) represents the exact result without Trotterization. (a) The orbital occupation number $\langle\Psi|\hat{a}_1^\dagger\hat{a}_1|\Psi\rangle$. (b) The fidelity with different Trotter steps Δt.

The time evolution of the fractional occupation number (FON) of ϕ_1, i.e. the spin-up configuration of the diabatic orbital η_a, is shown in Fig. S2(a) with different Trotter steps Δt. We can see the change of FON which indicates the electron transfer between fixed ions, since the orbitals are localized to the ions.

Figure S2(b) shows the fidelity $|\langle\Psi|\Psi_{\mathrm{exact}}\rangle|^2$ which indicates how exact the state $|\Psi\rangle$, the vibronic state propagated with Trotterization. The Trotter step converges approximately to the exact result at $\Delta t = 5.6$ a.u. ≈ 0.1 fs which is a reasonable time scale often used for traditional NAMD simulations. We can estimate the Trotter step size of an actual quantum simulation on a cMQB device from the ratio between time scales of molecular vibronic dynamics and the natural frequency of bosonic degrees of freedom in the cMQB device, which gives us $\Delta t \sim 0.1$ μs and ~ 0.1 ps for trapped ions and cQED, respectively. A longer timestep of $\Delta t = 11.2$ still quantitatively reproduces the dynamics of the FON, whereas $\Delta t = 22.4$ follows the dynamics closely for 1200 a.u.

We performed the cMQB simulation using the QuTiP package [3] in Python.

V. BORN-OPPENHEIMER FRAMEWORK SIMULATION

We can construct a full configuration interaction (FCI) space with three singlet configuration state functions (CSFs) for two electrons in four restricted spin orbitals, i.e. two closed shell configurations and one open-shell configurations:

$$|\Phi_1^{\mathrm{CSF}}\rangle = |1010\rangle, \tag{S14}$$

$$|\Phi_2^{\mathrm{CSF}}\rangle = |0101\rangle, \tag{S15}$$

$$|\Phi_3^{\mathrm{CSF}}\rangle = \frac{1}{\sqrt{2}}(|1001\rangle + |0110\rangle). \tag{S16}$$

The corresponding electronic Hamiltonian matrix in the CSF basis is,

$$H_{\mathrm{el}}^{\mathrm{CSF}} = \begin{pmatrix} 2h_{aa} + v_{aaaa} & v_{aabb} & \sqrt{2}(h_{ab} + v_{aaab}) \\ v_{aabb} & 2h_{bb} + v_{bbbb} & \sqrt{2}(h_{ab} + v_{bbba}) \\ \sqrt{2}(h_{ab} + v_{aaab}) & \sqrt{2}(h_{ab} + v_{bbba}) & h_{aa} + h_{bb} + v_{abab} + v_{aabb} \end{pmatrix}. \tag{S17}$$

Because all orbital vibronic couplings are zero in our model, the CSF basis itself is also a diabatic basis for the two-electron state. We obtain a Born-Oppenheimer (BO) basis set by diagonalizing the CSF Hamiltonian with a unitary transformation:

$$\Phi_j^{\mathrm{BO}}(r_1, r_2; R) = \sum_i \Phi_i^{\mathrm{CSF}}(r_1, r_2; R) U_{ij}(R). \tag{S18}$$

The electrons are localized around the left and right fixed ions for $|\Phi_1^{\mathrm{CSF}}\rangle$ and $|\Phi_2^{\mathrm{CSF}}\rangle$, respectively, while the open-shell singlet configuration $|\Phi_1^{\mathrm{CSF}}\rangle$ shows electron densities on the both side of the fixed ions. The joint electron-nuclear density function $\rho(r, R)$ of a two-electron state $\Phi(r_1, r_2; R)$ can be calculated as $2\int dr_1 \int dr_2\, \delta(r - r_1)|\Phi(r_1, r_2; R)|^2$. The joint density functions of CSF states and BO states are shown in Figs. S3 and S4, respectively. The BO states

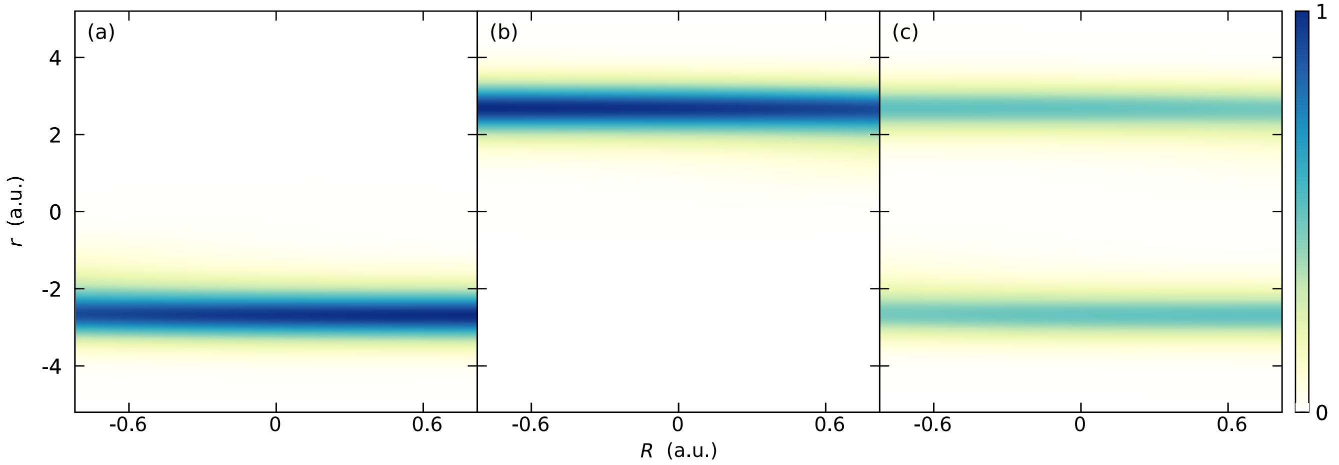

FIG. S3. The density function $\rho(r, R)$ for CSF states. (a) $|\Phi_1^{\mathrm{CSF}}\rangle$, (b) $|\Phi_2^{\mathrm{CSF}}\rangle$, and (c) $|\Phi_3^{\mathrm{CSF}}\rangle$.

show mixed character across the nuclear coordinate with a drastic change from one closed shell configuration to the other closed shell configuration at the origin for $|\Phi_2^{\mathrm{BO}}\rangle$ and $|\Phi_3^{\mathrm{BO}}\rangle$ indicating the strong nonadiabatic coupling (NAC) between them, while the coupling between $|\Phi_1^{\mathrm{BO}}\rangle$ and $|\Phi_2^{\mathrm{BO}}\rangle$ is relatively small. The diagonal CSF Hamiltonian matrix elements and the BO potential energy surfaces with nonadiabatic couplings (NACs) between BO states, $D_{ij} = \langle \Phi_i^{\mathrm{BO}} | \frac{\partial}{\partial R} \Phi_j^{\mathrm{BO}} \rangle$ using the coefficients in Table S.I are plotted in Fig. S5.

We performed the equivalent dynamics in the BO representation using the Born-Huang expansion [Eq. (11)], where the effective Hamiltonian in the BO representation for time-dependent Schrödinger equation can be found in Ref. 4. For the GBOA, the ground state $|\Phi_1^{\mathrm{BO}}\rangle$ is neglected since it has a relatively small NAC with the excited states compared to the NAC between the first and second excited states.

We used sinc discrete variable representation (DVR) for the nuclear basis on 1500 uniform grid points from $R = -1.0$ to 1.0 a.u. The initial state is prepared in the BO representation via the unitary transformation between the CSF and BO basis [Eq. (S18)] on the nuclear grid. The initial electronic state (corresponding to $|\Phi_1^{\mathrm{CSF}}\rangle$) has a negligible

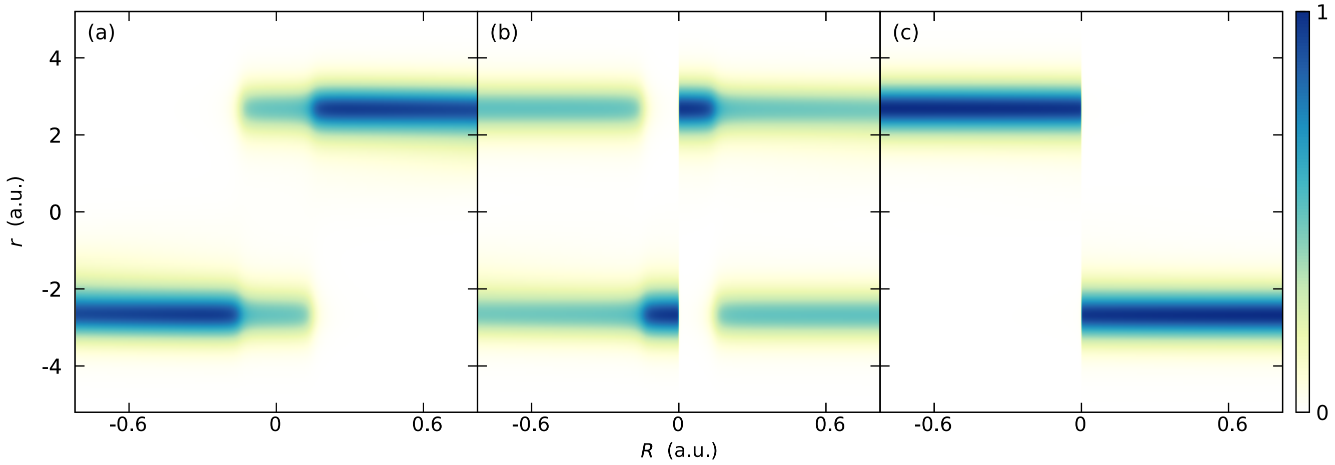

FIG. S4. The density function $\rho(r,R)$ for BO states: (a) $|\Phi_1^{\rm BO}\rangle$, (b) $|\Phi_2^{\rm BO}\rangle$, and (c) $|\Phi_3^{\rm BO}\rangle$.

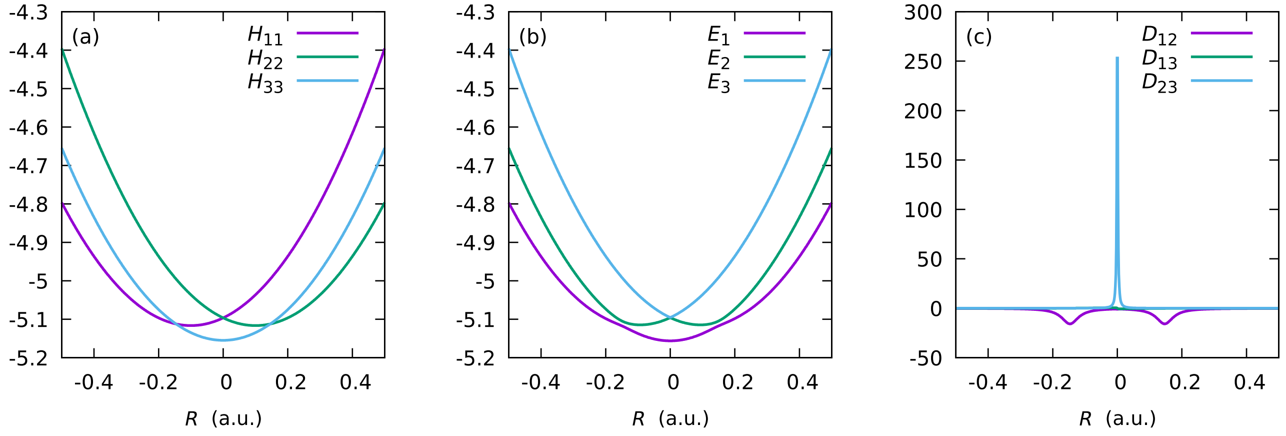

FIG. S5. (a) The diagonal CSF Hamiltonian matrix elements, (b) the BO potential energy surfaces, and (c) the NACs between BO states.

contribution on the BO ground state, thus the GBOA basis reproduces an accurate initial state. We propagated the wavefunction using a time-evolution matrix by direct exponentiation of the DVR Hamiltonian matrix in the BO representation.

We performed the BO framework simulation using the QuTiP package [3] in Python.

NOISE EFFECT

We first derive the superoperator and dissipation rate for indirect qubit noise from the vibrational decoherence in an ion trap device. The two-qubit entanglement can be achieved by a spin-dependent force using the laser-ion interaction with the Mølmer-Sørensen (MS) Hamiltonian,

$$\hat{H}_{\rm MS} = \frac{\Omega}{2}\hat{S}_x\left(\hat{b}e^{i\delta t} + \hat{b}^\dagger e^{-i\delta t}\right), \tag{S19}$$

where $\hat{S}_x = \hat{X}_p + \hat{X}_q$, Ω is the Rabi frequency, and δ is the detuning of the laser from the first sideband transition [5], which intermediately couples the qubit states and a motional mode of ions. After applying the MS interaction for time $\tau = \pi/\delta$ with $\delta = 2\Omega$, the ionic motion is decoupled from the qubit states while the two qubits become entangled by an effective two-qubit entanglement operation $R_{XX}(\pi/2) = \exp(-i\frac{\pi}{4}\hat{X}_p\hat{X}_q)$.

Next, we write the Lindblad master equation for the MS interaction with the vibrational decoherence in the interaction picture with respect to the MS Hamiltonian. The jump operator for the vibrational decoherence $\hat{n} = \hat{b}^\dagger\hat{b}$

becomes

$$\hat{L} = \hat{n} + \left(a(t)\hat{b}^\dagger + a^*(t)\hat{b}\right)\hat{S}_x + |a(t)|^2\hat{S}_x^2, \tag{S20}$$

where $a(t) = -i\frac{\Omega}{2}\int^t e^{-i\delta t'}dt' = \frac{e^{-i\delta t}-1}{4}$. We trace over the motional degrees of freedom to obtain the master equation for reduced density operator for the qubits assuming $\tau \ll \frac{1}{\gamma^{\rm d}_{\rm mot}}$ [6]

$$\begin{aligned}\frac{d}{dt}\hat{\rho}_{\rm q} &\approx \gamma^{\rm d}_{\rm mot}\langle|a(t)|^2\rangle_\tau\left(2\langle\hat{n}\rangle+1\right)D[\hat{S}_x]\hat{\rho}_{\rm q} + \gamma^{\rm d}_{\rm mot}\langle|a(t)|^4\rangle_\tau D[\hat{S}_x^2]\hat{\rho}_{\rm q}\\ &\approx \frac{2\langle\hat{n}\rangle+1}{8}\gamma^{\rm d}_{\rm mot}D[\hat{S}_x]\hat{\rho}_{\rm q}\\ &\equiv \gamma^{\rm q}D[\hat{S}_x]\hat{\rho}_{\rm q}\end{aligned} \tag{S21}$$

where $\gamma^{\rm d}_{\rm mot}$ is the motional decoherence rate, $\langle\hat{n}\rangle$ is the initial expectation value of the number operator of the motional mode, and $\langle f(t)\rangle_\tau \equiv \int^\tau f(t)dt/\tau$ is the time average of the function f over time τ. We neglect the second term in the first line of the above equation since it is negligible compared to the first term.

In order to map the motional decoherence noise in an ion trap device onto the molecular dynamics, we have to scale the rate as $\gamma^i_{\rm mol} = \alpha_i\gamma^{\rm d}_{\rm mot}$ where $i = \{\text{vib}, \text{q}\}$ represent the corresponding degrees of freedom of the molecule. The scaling factor connects the molecular and simulation scales: $\hat{H}_{\rm sim} = F\hat{H}_{\rm mol}$, where the maximum value $F^{\rm max}$ is chosen usually to make $t_{\rm sim}$ the smallest possible for reliable simulation within the shortest coherence time. Since the time subjected to the bosonic decoherence noise for bosonic modes and for qubits via the bus mode differ, their scaling factors are also different. First, the bosonic modes for nuclear degrees of freedom are subjected to bosonic noise for the entire experiment time for a cMQB simulation,

$$t_{\rm exp} = (t_{\rm CNOT}N_{\rm CNOT} + \Delta t_{\rm sim}N_{\rm op})N_t. \tag{S22}$$

Thus, the scaling factor becomes

$$\alpha_{\rm vib} = \frac{t_{\rm exp}}{t_{\rm mol}} = N_{\rm CNOT}\frac{t_{\rm CNOT}}{\Delta t_{\rm mol}} + \frac{N_{\rm op}}{F}. \tag{S23}$$

On the other hand, a pair of qubits is subject to the indirect noise from the bus mode only when an entanglement operator is applied to those qubits, which yields

$$\alpha_{\rm q} = \frac{t_{\rm CNOT}}{\Delta t_{\rm mol}}, \tag{S24}$$

for a single entanglement operator. Since two qubits are subjected to the noise per CNOT gate, the effective rate for a single qubit can be calculated approximately by multiplying the number of qubits subjected to CNOT gates divided by the total number of qubits,

$$\alpha^{\rm eff}_{\rm q} = \frac{2N_{\rm CNOT}}{N_{\rm q}}\alpha_{\rm q}. \tag{S25}$$

Since the vibrational bus mode can be efficiently cooled to the motional ground state [7] during initialization in an ion trap,

$$\gamma^{\rm q} \approx \frac{1}{8}\gamma^{\rm d}_{\rm mot}. \tag{S26}$$

Finally, we get Eqs. 9 and 10.

Noise simulation

Adding a constant value to all orbital energies h_{pp} does not change the dynamics because h_{pp} only appears as the coefficient of a single qubit operator as $h_{pp}\hat{n}_p$. Thus, adding a constant only results in global phase change

$$\sum_{\mathbf{n}}\exp\left(-it\sum_p(h_{pp}+c)\hat{n}_p\right)|\mathbf{n}\rangle = \exp(-itcN_{\rm e})\sum_{\mathbf{n}}\exp\left(-it\sum_p h_{pp}\hat{n}_p\right)|\mathbf{n}\rangle. \tag{S27}$$

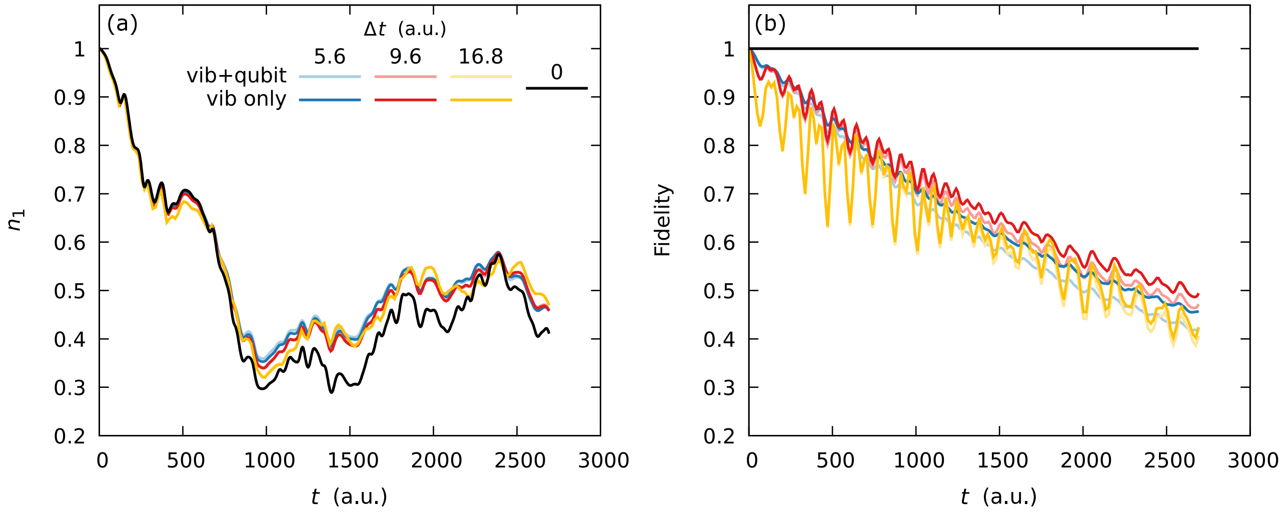

FIG. S6. Comparison of noise effects due to motional decoherence with different Trotter step sizes for cMQB simulations. (a) Time-evolution of the fractional occupation number of ϕ_1. (b) Time-evolution of the fidelity of the molecular wavefunctions The blue, red, and green lines represent the noise simulation results with $\Delta t = 5.6$, 9.6, and 16.8 a.u., respectively, where the light colors represent the corresponding simulation results with both bosonic and qubit noise and the black line represents the exact closed system simulation result without Trotterization.

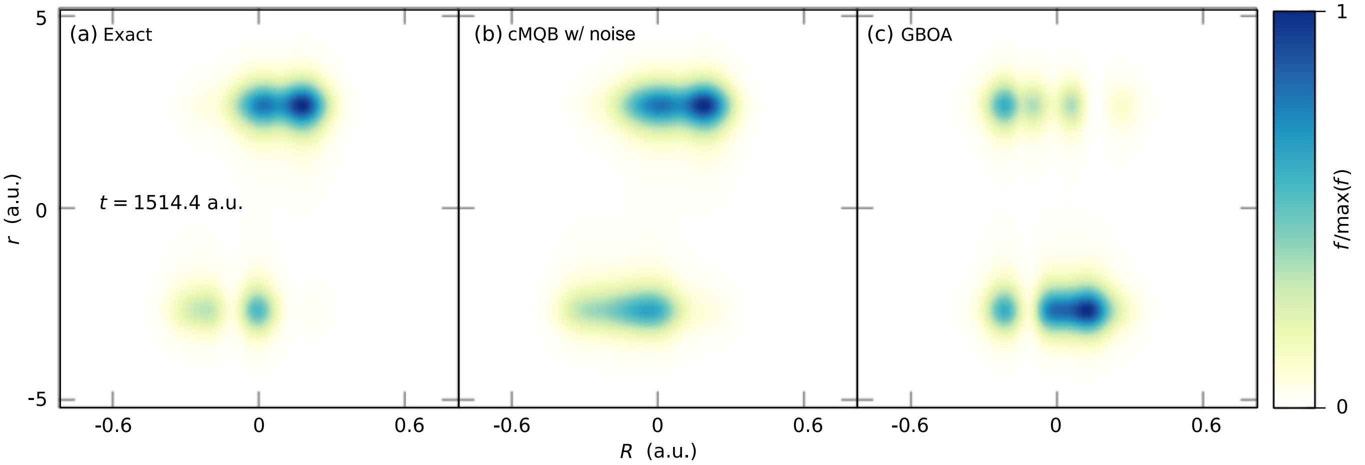

FIG. S7. The density functions $\rho(r, R, t)$ at, $t = 1514.4$ a.u., with (a) exact time-evolution of closed system, (b) Trotterized cMQB time-evolution with noise effect ($\Delta t = 5.6$ a.u.), and (c) GBOA for closed system. Spatial functions are normalized to their maximum values.

Therefore, in our model case, the orbital energies can be set to zero since they have same values. The largest value remaining is $v_{aaaa} = v_{bbbb} = 0.2236$ a.u. (Table. S.I) which yields the scaling factor $F = \dfrac{10^6\text{Hz} \times 1.51983 \times 10^{-16}\ \text{a.u./Hz}}{0.2236\ \text{a.u.}} \sim 6.8 \times 10^{-10}$ assuming a 1 MHz maximum Rabi frequency [8]. From the given Rabi frequency, we can calculate the entanglement time $t_{\text{CNOT}} \sim 1.57 \times 10^{-6}$ s and we used the value $\gamma^{\text{d}}_{\text{mot}} = 30\ \text{s}^{-1}$ [9] for the bosonic coherence time. Finally, we have $N_{\text{op}} = 33$ and $N_{\text{CNOT}} = 76$ for our model [Eq. (S13)].

We performed the open quantum system simulations for the noise effect using the QuTiP package [3] in Python.

[1] S. Shin and H. Metiu, Nonadiabatic effects on the charge transfer rate constant: A numerical study of a simple model system, J. Chem. Phys. **102**, 9285 (1995).

[2] Y. Suzuki and K. Yamashita, Real-time electron dynamics simulation of two-electron transfer reactions induced by nuclear motion, Chem. Phys. Lett. **531**, 216 (2012).

[3] J. Johansson, P. Nation, and F. Nori, QuTiP 2: A Python framework for the dynamics of open quantum systems, Comput. Phys. Commun. **184**, 1234 (2013).

[4] G. A. Worth and L. S. Cederbaum, Beyond Born-Oppenheimer: Molecular Dynamics Through a Conical Intersection, Annu. Rev. Phys. Chem. **55**, 127 (2004).

[5] K. Mølmer and A. Sørensen, Multiparticle Entanglement of Hot Trapped Ions, Phys. Rev. Lett. **82**, 1835 (1999).

[6] F. Haddadfarshi and F. Mintert, High fidelity quantum gates of trapped ions in the presence of motional heating, New J. Phys. **18**, 123007 (2016).

[7] C. H. Valahu, V. C. Olaya-Agudelo, R. J. MacDonell, T. Navickas, A. D. Rao, M. J. Millican, J. B. Pérez-Sánchez, J. Yuen-Zhou, M. J. Biercuk, C. Hempel, T. R. Tan, and I. Kassal, Direct observation of geometric-phase interference in dynamics around a conical intersection, Nat. Chem. **15**, 1503 (2023).

[8] T. R. Tan, J. P. Gaebler, R. Bowler, Y. Lin, J. D. Jost, D. Leibfried, and D. J. Wineland, Demonstration of a dressed-state phase gate for trapped ions, Phys. Rev. Lett. **110**, 263002 (2013).

[9] V. C. Olaya-Agudelo, B. Stewart, C. H. Valahu, R. J. MacDonell, M. J. Millican, V. G. Matsos, F. Scuccimarra, T. R. Tan, and I. Kassal, Simulating open-system molecular dynamics on analog quantum computers, arXiv [quant-ph]:2407.17819 (2024), arXiv:2407.17819.
